# Whole Genome Sequencing and Metabolomic Study of Cave *Streptomyces* Isolates ICC1 and ICC4

**DOI:** 10.3389/fmicb.2019.01020

**Published:** 2019-05-10

**Authors:** Jessica Thandara Gosse, Soumya Ghosh, Amanda Sproule, David Overy, Naowarat Cheeptham, Christopher N. Boddy

**Affiliations:** ^1^Department of Chemistry and Biomolecular Sciences, University of Ottawa, Ottawa, ON, Canada; ^2^Department of Biological Sciences, Faculty of Science, Thompson Rivers University, Kamloops, BC, Canada; ^3^Ottawa Research and Development Centre, Agriculture and Agri-Food Canada, Ottawa, ON, Canada

**Keywords:** *Streptomyces*, whole genome sequencing, secondary metabolites, genome, metabolome

## Abstract

The terrestrial subsurface microbiome has gained considerable amount of interests in the recent years because of its rich potential resource for biomining novel genes coding for metabolites possessing antimicrobial activities. In our previous study, we identified two *Streptomyces* isolates, designated as ICC1 and ICC4, from the Iron Curtain Cave, Chilliwack, Canada that exhibited antagonistic activities against the multidrug resistant strains of *Escherichia coli*. In this study, the genomes of these two isolates were sequenced by Illumina MiSeq, assembled and annotated. The genes associated with secondary metabolite production were identified and annotated using the bioinformatics platforms antiSMASH and BAGEL. ICC1 and ICC4 were then cultivated and ICC1 metabolome characterized by UHPLC-ESI-HRMS. The Global Natural Products Social Molecular Networking was used to identify metabolites based on the MS/MS spectral data. ICC1 and ICC4 showed a high level of sequence identity with the terrestrial bacteria *Streptomyces lavendulae*; however, they possess a greater secondary metabolite potential as estimated by the total number of identified biosynthetic gene clusters (BGCs). In particular, ICC1 and ICC4 had a greater number of polyketide and non-ribosomal peptide BGCs. The most frequently detected BGCs were those predicted to generate terpenes, small and low complexity dipeptides and lipids. Spectral analysis clearly identified a number of diketopiperazine products through matched reference spectra for cyclo (Leu-Pro), cyclo (Pro-Val) and cyclo [(4-hydroxyPro)-Leu]. One of the terpenes gene clusters predicted by antiSMASH possesses a seven-gene pathway consistent with diazepinomicin biosynthesis. This molecule contains a very rare core structure and its BGC, to date, has only been identified from a single bacterial genome. The tetrapeptide siderophore coelichelin BGC was unambiguously identified in the genome, however, the metabolite could not be identified from the culture extracts. Two type III polyketides, 2′, 5′ – dimethoxyflavone and nordentatin, were identified from the UHPLC-HRMS data of the aqueous and *n*-butanolic fractions of *Streptomyces* sp. ICC1, respectively. A BGC likely encoding these metabolites was predicted in both genomes. The predicted similarities in molecule production and genome shared by these two strains could be an indicative of a cooperative mode of living in extreme habitats instead of a competitive one. This secondary metabolite potential may contribute to the fitness of ICC1 and ICC4 in the Iron Curtain Cave.

## Introduction

Microbial life is ubiquitous in the Earth’s crust. As early as 1980s, the presence of viable microbes were found in various shallow aquifers, caves, and mines, among other subsurface environments (Ghiorse and Wilson, 1988; Yates et al., 1988; Lovley, 1997). These microorganisms have drawn a considerable amount of interest because of their wide distribution in the terrestrial subsurface and their influence on geochemical process, quality of underground water, and transport of environmental contaminants (Balkwill et al., 1997). Although many subsurface environments are challenging to access, representative data is obtained from samples collected from mines and caves (Bonis and Gralnick, 2015; Kutvonen et al., 2015; Miettinen et al., 2015; Rajala et al., 2015), from water seeping through mine walls (Borgonie et al., 2015), by collecting rock material and scrapings from cave (Wu et al., 2015) and mines surfaces (Dziewit et al., 2015).

Studies of such environments have shown that metals play a pivotal role in determining subsurface living communities (Parnell et al., 2015). For instance, iron is one of the most abundant metals in the Earth’s crust and is present in various biomolecules (Parnell et al., 2015). Iron is essential for the function of many fundamental, conserved biochemical processes, such as electron transport and the Krebs cycle (Bonis and Gralnick, 2015). Many cave bacterial species use iron in a variety of ways, such as the genus *Thiobacillus*, which can obtain energy by oxidizing sulfur and ferrous iron compounds (De Mandal et al., 2017). Iron ore caves contain a variety of microbial taxa with member species capable of dissimilatory iron reduction, including the Chloroflexi, Acidobacteria and the Alpha-, Beta- and Gammaproteobacteria. Their biological activity, combined with mass transport of solubilized Fe(II) by groundwater, contribute to speleogenesis of banded iron formations (Parker et al., 2018).

Caves represent the hidden earth (Taylor, 1999; Culver and Pipan, 2009; Cheeptham, 2013; Ghosh et al., 2016). Caves are subsurface habitats that possess unique characteristics including limited amounts of light, low amounts of organic nutrients (depending on the cave), higher humidity, and a higher concentration of minerals (Cheeptham, 2013). These diverse features create microenvironments, some of which are further modified by the microbial processes occurring within them (Barton and Northup, 2007; Montano and Henderson, 2013). The cave subsurface microbial diversity is specific in their association to the rock surfaces and influenced by the rock’s chemistry and the organism’s metabolic requirements and tolerances (Boston et al., 2001; Northup et al., 2011). Adaptive microbes thrive in these extreme conditions (Barton, 2006, 2015; Barton and Northup, 2007; Ghosh et al., 2016) because of their unique physiology and exploitation of different and specialized metabolic pathways (Cheeptham et al., 2013). These adaptations in such extreme nutrient-limited environments often lead to the production of various secondary metabolites and the acquisition of a unique antibiotic resistome necessary for their survival (Bhullar et al., 2012; Gabriel and Northup, 2012). These genes make such organisms excellent candidates for new antibiotic and enzyme discovery for potential industrial development (Montano and Henderson, 2013; Ghosh et al., 2016). For instance, a previous study (Bhullar et al., 2012) at the Lechuguilla Cave in New Mexico identified over 500 distinct microbial isolates from a region of the cave thought to be isolated from surface input for over 4 million years. Of these strains, 96 exhibited resistance toward at least one of 26 different commercially available antibiotics. Many showed resistance to multiple antibiotics. Sequencing of one of these isolates showed *mphE*, which encodes resistance to the macrolide antibiotics, that shared 72% identity with a homolog from a terrestrial soil isolate (Bhullar et al., 2012).

Microbial diversities from cave habitats have shown that cave bacteria, such as the cultivable actinomycetes, possess an immense pool of secondary metabolites (Maciejewska et al., 2016, 2017; Ghosh et al., 2017). Among the actinomycetes, bacteria of the genus *Streptomyces* are the most prolific producers of secondary metabolites, many of which have interesting antimicrobial potential (Cheeptham et al., 2013; Kumar et al., 2014; Lee et al., 2014; Nimaichand et al., 2015; Axenov-Gribanov et al., 2016; Hamm et al., 2017; Adam et al., 2018;Maciejewska et al., 2018).

Advances in next-generation sequencing technology and analysis over the past 15 years have revolutionized microbial genomics and our ability to unravel a microorganism’s metabolic potential through bioinformatic analyses (Gomez-Escribano et al., 2016). Comprehensive bioinformatic tools for genome-wide detection and annotation of secondary metabolite biosynthetic gene clusters (BGCs), like the web tool antiSMASH 3.0 (Weber et al., 2015) and the BGC repository MIBiG (Medema et al., 2015), have resulted in an unprecedented rate of new natural product biosynthetic pathway discovery. These tools are enabling a more detailed understanding of bacterial metabolism and physiology.

Previously we cultivated and identified two *Streptomyces* isolates, designated as ICC1 and ICC4, from the Iron Curtain Cave in Chilliwack, Canada (Ghosh et al., 2017). Extracts from these strains exhibited antagonistic activities against multidrug resistant bacterial strains of *Escherichia coli* and non-resistant strain of *Staphylococcus aureus* when grown in Hickey–Tresner (HT) and V8 broth media and incubated at 8 and 15°C for 10 days on a rotatory shaker (≈200 rpm) (Ghosh et al., 2017). Herein we describe the sequencing and annotation of the genomes of these strains and profile their metabolome to identify secondary metabolites. While the genomes of the isolates show high sequence identity to the terrestrial strain *Streptomyces lavendulae*, they differ in their secondary metabolite biosynthesis.

## Materials and Methods

### ICC1, ICC4 Cultivation and Whole Genome Sequencing

ICC1 and ICC4 were cultivated as per a previous study (Ghosh et al., 2017). ICC1 and ICC4 were cultivated on R2A (HiMedia Laboratories Pvt. Ltd., Nashik, India) or Hickey–Tresner (HT) (Yeast extract 0.1%, Beef extract 0.1%, N-Z-Amine 0.2%, Dextrin 1%, pH 7.3) (Cheeptham et al., 2013) broth media. Following the incubation in R2A for 7 days, genomic DNA was extracted using PROMEGA Wizard Genomic DNA Extraction Kit (PROMEGA, United States) following the manufacturer’s instructions.

A preliminary identification of the organisms was performed by 16S rRNA amplification and sequencing. ICC1 and ICC4 genomic DNA was sent for whole genome sequencing using WGS services from the Centre for Comparative Genomics and Evolutionary Bioinformatics – Dalhousie University. The library for each sample was prepared using a Nextera DNA sample preparation kit (Illumina) following the manufacturer’s instructions. The library was sequenced using a 600-cycle v3 reagent kit (Illumina), with an average sequencing coverage of 3.8×. The raw files were assembled using PATRIC Genome Assembly Service and annotated using RAST (Rapid Annotation using Subsystem Technology) online tools (Wattam et al., 2017). Genomes are available on the NCBI database (Accession numbers CP030286 and CP030287).

### Bacterial Metabolites Extract Generation and Solvent Fractioning

ICC1 and ICC4 were cultivated to identify secondary metabolites produced and to screen their extracts for antimicrobial activities. Four hundred milliliters of both strains were cultured as per our previous study (Ghosh et al., 2017). Ten milliliters of each bacterial culture were collected weekly to monitor their secondary metabolite production profile.

After a month of cultivation in HT liquid media, the fermented broth was collected and centrifuged at 4000 rpm for 15 min and filtered using a 0.22 μm syringe filter. From the filtrate, 50 mL were taken for solvent fractionation and the remaining broth was frozen at –20°C for future use. The aliquot was freeze-dried and resuspended in 10 mL methanol for solvent fractionation using a modified Kupchan partition method (Kupchan et al., 1973).

Water (1 mL) was added to the methanol fraction to adjust the aqueous content to 10% *v*/*v.* An equal volume of *n*-hexane (11 mL) was added and the solution was vigorously shaken. After phase separation, the upper phase was collected and the *n*-hexane was removed through rotary evaporation, generating the *n*-hexane fraction. The remaining methanolic phase had the aqueous content adjusted to 40% water *v*/*v* (approx. 3 mL of water). To this, an equal volume of chloroform (14 mL) was added, vigorously shaken and after phase separation the organic lower layer was collected and evaporated to dryness, generating the chloroform fraction.

The remaining liquid phase was stripped of traces of methanol and chloroform via evaporation and reconstituted to its original volume (10 mL) by the addition of water. An equal volume of *n*-butanol (10 mL) was added, vigorously shaken, and upon phase separation the upper fraction was collected and the solvent evaporated to generate the butanol fraction. The remaining aqueous phase was concentrated to remove traces of *n*-butanol and freeze-dried, generating the aqueous fraction.

### Antibiotic Activity Assays

All filter sterilized culture samples and Kupchan partition fractions were tested for antibacterial activity against a set of microorganisms by the Kirby-Bauer disk diffusion assay, as briefly described. Filter paper disks were prepared using a hole punch on Grade 3 Whatman filter papers. The disks were autoclaved and were soaked with 20 μL of the desired testing solutions. The disks were air dried prior to use. Antibiotic controls were prepared according to CLSI guideline standards (National Committee for Clinical and Laboratory Standards Institute, 2012). Bacteria to be tested were prepared following CLSI standards. *E. coli* BW25113, *Pseudomonas aeruginosa* PA01, and *Bacillus subtilis* 168 were grown overnight at 37°C in Mueller-Hinton broth from single colonies. Overnight cultures were diluted in Mueller-Hinton broth to reach OD600 between 0.07 and 0.1. The bacterial culture was spread evenly on Petri dishes containing Mueller-Hinton agar using a cotton swab and were left to dry for a short period of time (5 min). Disks containing testing solutions and controls were placed onto the prepared agar. Plates were incubated for 4 to 6 days at 15°C and examined for the presence or absence of inhibition halos.

### Solid Phase Extraction

Fractions that exhibited antibiotic activity in disk diffusion assays were further fractionated by solid phase extraction (SPE) using a positive pressure manifold to generate subfractions for further analysis. Approximately 5 mg of each active fraction generated by Kupchan partition were resuspended in 1 mL of water and applied to a 1000 mg HyperSep C18 cartridge (Thermo Fisher Scientific, United States). The flow-through was collected as subfraction A. Subfraction B was obtained by washing the sample vial with 100 μL of 1:1 acetonitrile:water, adding the wash to the cartridge and eluting with 1 mL of the same solvent. Elution with 1 mL of acetonitrile followed by 1 mL of 1:1 methanol:dichloromethane generated subfractions C and D, respectively. All subfractions were evaporated to dryness.

### UHPLC-HRMS Analysis

Subfractions A and B were resuspended in 1:1 acetonitrile:water to a concentration of 500 μg/mL while C and D were resuspended in acetonitrile to the same concentration. The original Kupchan partition fractions were also resuspended in 1:1 acetonitrile:water (aqueous fraction) or 1:1 acetonitrile:methanol (*n*-hexanes, chloroform and *n*-butanol fractions). A 5 μL aliquot was analyzed using a Thermo Fisher Scientific Dionex Ultimate 3000 UHPLC system coupled to a Thermo LTQ Orbitrap XL high resolution mass spectrometer. Chromatography was performed using a Phenomenex Kinetex C18 100 Å column (2.1 × 50 mm, 1.7 μm) with a flow rate of 0.35 mL/min. The mobile phase consisted of water containing 0.1% formic acid (solvent A) and acetonitrile containing 0.1% formic acid (solvent B). The gradient began at 5% solvent B, increased to 95% over 4.5 min and remained constant at 95% for 3.5 min. The mobile phase returned to starting conditions over 0.5 min and was left to equilibrate for 6 min. The HRMS was operated in ESI^+^ mode using the following parameters: sheath gas (40), auxiliary gas (5), sweep gas (2), spray voltage (4.0 kV), capillary temperature (320°C), capillary voltage (35 V), tube lens (100 V), maximum injection time (500 ms) and microscans (1). A full MS1 scan (*R* = 30000) from *m/z* 50–2000 was performed in the Orbitrap for both the ICC1 and ICC4 extracts and in the case of the ICC1 extracts the top 6 most intense ions in each scan were fragmented using collision induced dissociation (35 eV) and analyzed sequentially by an MS2 scan in the low-resolution ion trap. RAW files were converted to .mzXML using RAWConverter (He et al., 2015) to be suited for further spectral analysis.

### Spectral Networking Analysis

The MS analysis used the Capture Compound Mass Spectrometry Technology ProteoSAFe Workflow Input Form. No Data Preset was chosen. Speclibs was the spectral library used as a comparison parameter. Multiple input MS/MS spectra for the raw fractions and subfractions in .mzXML formats were uploaded the same. Precursor Mass tolerance and Fragment Ion Mass tolerance were set as 2.0 and 0.5 Da, respectively. Advanced parameters were set as standards. Results were analyzed based on *m/z* errors and mass difference.

## Results

### Activity Assays

Fractions originated from Kupchan partition showed activity against all strains tested. Disk diffusion assays for ICC1 showed activity of the *n*-butanol fraction against *E. coli* BW25113 and the aqueous fraction against *B. subtilis* 168. *P. aeruginosa* PA01 was susceptible to the unfractionated filtered broth of strain ICC1.

### Whole Genome Sequencing and Assembly of ICC1 and ICC4

Preliminary 16S rDNA sequencing of ICC1 and ICC4 indicated that both organisms belong to the genus *Streptomyces*, which is supported by morphology and growth characteristics of the bacteria. Whole genome sequencing by Illumina MiSeq (Illumina, United States) and *de novo* genome assembly by PATRIC (Wattam et al., 2017) generated draft genomes of 9,034,309 bp for ICC1 and 9,010,404 bp for ICC4, with a G+C content of 72% for both strains. The ICC1 genome was assembled into 726 contigs and the ICC4 genome into 734 contigs with N50s of 22,631 and 23,307, respectively. RAST annotation predicted 8235 coding sequences for ICC1 and 8243 coding sequences for ICC4. Sequences were deposited in the NCBI database (Accession numbers CP030286 and CP030287).

### Comparative Genomic Analysis

**FIGURE 1:**
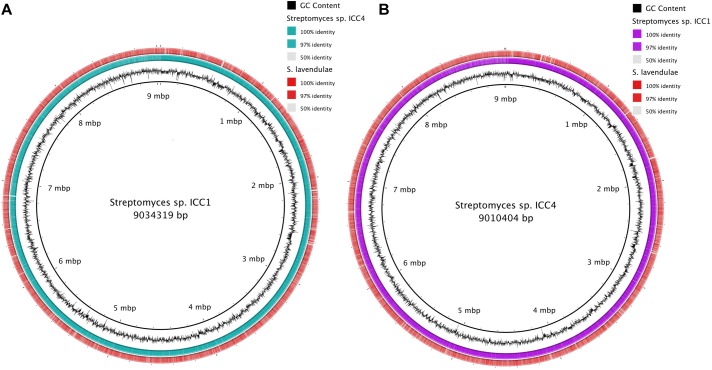
Genome alignment of genomes of *Streptomyces* sp. **(A)** Alignment of *Streptomyces* sp. ICC1 against *Streptomyces* sp. ICC4 and *Streptomyces lavendulae* strain CCM 3239. **(B)** Alignment results of *Streptomyces* sp. ICC4 against *Streptomyces* sp. ICC1 and *S. lavendulae* strain CCM 3239.

Genomic comparison files were generated online at WebACT (Abbott et al., 2005) and functional analysis was performed using Artemis Comparison Tool (ACT; Carver et al., 2005). Strains were aligned using BLAST to compare genomic profiles between each other and with other genomes on the database. The closest genome match to both ICC1 and ICC4 was *S. lavendulae* strain CCM 3239 (accession number CP024985), with alignments of 54% of the sequence for both ICC1 and ICC4 and identities of 90% within both of the alignment (Figure 1). The average nucleotide identity for both ICC1 and ICC4 with *S. lavendulae* was 87.9%. When compared to each other, the genomes of ICC1 and ICC4 showed 50% coverage with 99% identity, indicating that the two strains are highly related.

**FIGURE 2:**
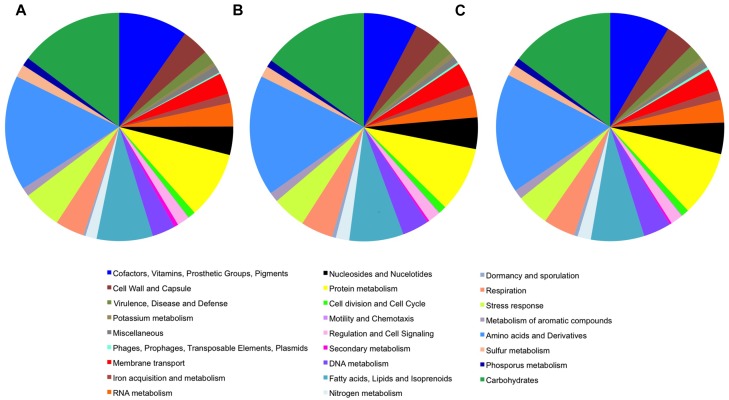
Genome features of *Streptomyces* sp. **(A)** *Streptomyces lavendulae* strain CCM 3239. **(B)** *Streptomyces* sp. strain ICC1. **(C)** *Streptomyces* sp. strain ICC4.

Analysis of the features of the cave *Streptomyces* genomes with those of *S. lavendulae* using RAST annotation revealed very similar predicted protein content and function between the organisms (Figure 2). *Streptomyces* sp. ICC1 and *Streptomyces* sp. ICC4 have a total of 450 subsystems while *S. lavendulae* has 435, indicating all three strains likely possess similar specific biological processes and structural complexes (Overbeek et al., 2005). Examination of the subsystem feature counts shows that all three strains have similar numbers of gene features in each subsystem, with *S. lavendulae* showing more complete features involved in secondary metabolite production (22) than the cave strains (12). These data are strongly supportive of all three strains being highly related.

### Secondary Metabolite Profiling and Genome Mining

The genomes of ICC1 and ICC4 were analyzed for the presence of secondary metabolite BGCs using antiSMASH 4.1.0 and BAGEL4 (van Heel et al., 2018). AntiSMASH 4.1.0 uses Hidden Markov Models (HMM) and rules based-detection to identify a broad array of BGCs, including those encoding polyketide, non-ribosomal peptides, terpenes, aminoglycosides and ribosomally synthesized and post-translationally modified peptides (RiPPs) from bacterial genomes. Similarly BAGEL4 uses HMM to detect core RiPP encoding genes, however, it is independent from the genome ORF calls, enabling it to better detect the small precursor peptides found in RiPP encoding gene clusters.

**Table 1:** Summary of antiSMASH 4.1.0 secondary metabolite prediction of *Streptomyces* sp. strains ICC1 and ICC4.

Metabolite Class	ICC1 Clusters	ICC4 Clusters	Most similar pathway (%)
PKS I	1	2	Meilingmycin (3%)
PKS II	2	2	Simocyclinone (66%)
PKS III	1	1	Alkylresorcinol (100%)
NRPS	5	3	Coelichelin (100%)
Siderophore	2	–	Salinomycin (4%)
Lantipeptide	2	2	SapB (100%)
Butyrolactone	4	3	Calcimycin/Salinomycin (10%)
Thiopeptide	–	1	Rifamycin (5%)
Melanin	2	2	Melanin (100%)
Bacteriocin	–	2	–
Terpene	10	10	2-methylisoborneol (100%)
Mixed	3	2	Kinamycin (8%)
Other	4	4	BE-14106 (14%)
TOTAL	37	35	

AntiSMASH predicted 37 putative secondary metabolite clusters for ICC1 and 35 for ICC4 (Table 1). The majority of these results were terpene BGCs, which presented low overall similarity with known natural products (under 20%); suggesting that these pathways may encode new natural products or natural products with no characterized BGCs. Both strains have in common a non-ribosomal peptide biosynthetic pathway with 100% similarity to coelichelin, a ferric iron-chelating peptide (Ziemert et al., 2016) and two lantipeptides, the morphogenic peptide SapB (Marsh et al., 2010) and an unidentified molecule (due to the lack of a predicted peptide core). Strain ICC4 has a unique thiopeptide prediction, which is particularly interesting due to its low similarity with other known BGC. It shows only 5% similarity to rifamycin suggesting that it may encode new chemical diversity.

To further characterize the unidentified lantipeptide BGCs, both genomes were analyzed with BAGEL (Hart and Moffat, 2016), a bioinformatic tool that specializes in the detection and annotation if RiPPs such as lantipeptides. BAGEL predicted the same lantipeptides identified by antiSMASH and did not provide further insight into the unidentified lantipeptides BGCs. BAGEL predicted an additional RiPP gene cluster in ICC1 and ICC4, which was assigned as the glyocin BGC based on sequence similarity. Interestingly the genes for glyocin production were observed within the melanin clusters in both cave strains and seem to be involved in the production and glycosylation of bacteriocins (Aydillo et al., 2014).

A detailed manual analysis of the genomes for BGC prediction revealed two independent BGCs containing the pyoverdin synthesis genes and a non-ribosomal peptide synthetases (NRPS) that was not identified by antiSMASH prediction from 6371985 to 6374537 bp in the *Streptomyces* sp. ICC1 genome. The NRPS alignment detected a *Streptomyces* multispecies synthase with 99% coverage and 81% similarity. A PKS-NRPS mixed cluster was also identified from 6080168 to 6149842 bp, however this is likely a false positive as the BLAST alignment and annotation shows the protein coding sequences as belonging to an uncharacterized protein. The assignment was further confirmed by UniProt and Pfam analysis.

### Correlating the Metabolome and Genome

To characterize the metabolic profile of these two strains, they were cultivated in V8 media for 30 days at 15°C and the spent culture broths were processed by the Kupchan partition to generate four fractions per strain, which were further sub-fractionated by a polarity-guided solvent gradient elution from C18 SPE resin. UHPLC-HRMS analysis enabled the identification of a large number of metabolites from the fractions exhibiting antimicrobial activity. In total 67 compounds (Supplementary Table S1) from ICC1 were identified through Global Natural Products Social Molecular Networking (GNPS; Wang et al., 2016), the majority of spectra matching with submitted spectra from Bronze datasets in GNPS. Of these, 26 are likely contaminants such as plasticizers and detergents or trivial compounds, such as arginine. The overall metabolite profile showed a large number of lipids and aromatics such as 5-aminovaleric acid, palmitamide and the glucoside 2,4-dihydroxy-7-methoxy-1,4-benzoxazin-3-one. Several cyclic and acyclic dipeptides were identified, with proline, histidine and isoleucine being the most common residues.

**FIGURE 3:**
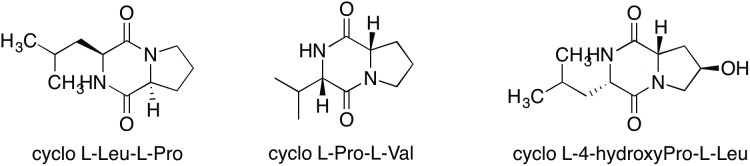
Diketopiperazines identified from *Streptomyces* sp. strain ICC1 by GNPS analysis of UHPLC-HRMS analysis of culture extracts.

Some metabolites could not be readily correlated to BGCs. For example GNPS spectral analysis clearly identified a number of diketopiperazine products from ICC1. Spectra matched reference spectra for cyclo (L-Leu- L-Pro), cyclo (L-Pro-L-Val) and cyclo (L-(4-hydroxyPro)-L-Leu) (Figure 3) from Bronze datasets in the GNPS database with low mass error. While these diketopiperazines were detected, their absolute stereochemistry remains unassigned. No corresponding NRPS or cyclodipeptide synthase encoding genes were identified in the genome. As these diketopiperazines are known to exhibit antifungal activity (Kumar and Nambisan, 2014) and likely play a role in the chemical ecology of the organism, future identification of their BGCs is an important task.

**FIGURE 4:**
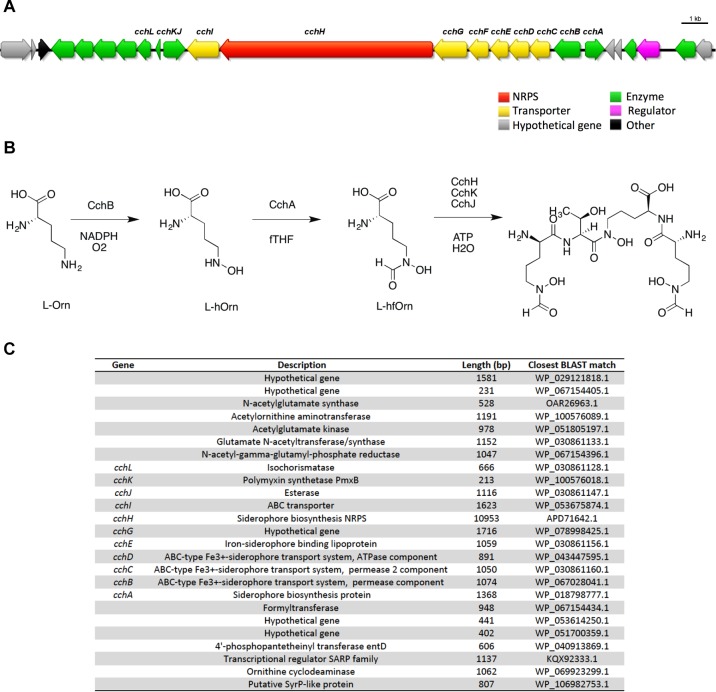
Coelichelin, one of the secondary metabolites predicted in *Streptomyces* sp. cave isolates. **(A)** Biosynthetic gene cluster for coelichelin. **(B)** Proposed mechanism for biosynthesis. **(C)** List of genes in the cluster, their annotation, length and similarity based on BLAST library alignment.

In some cases a BGC was unambiguously identified and assigned to a known compound, however, the metabolite was not detected from the culture extracts. For example, antiSMASH identified a NRPS trimodular gene cluster with 100% similarity to the tetrapeptide siderophore coelichelin from the genomes of ICC1 and ICC4 (Figure 4). None of the fractions analyzed by UHPLC-HRMS showed evidence for the presence of coelichelin. The coelichelin gene cluster was first sequenced and characterized from *Streptomyces coelicolor* (Lautru et al., 2005); soil-dwelling bacteria responsible for breaking down organic matter under variable environmental conditions. As many siderophores are produced only under iron-limiting conditions (Bagg and Neilands, 1987; D’Onofrio et al., 2010) and *Streptomyces* sp. strains ICC1 and ICC4 were cultivated under non-iron limiting conditions, it is possible that the gene clusters remained silent and unexpressed in our experimental conditions.

A number of metabolites identified by UHPLC-HRMS followed by GNPS analysis could be correlated with BGCs identified by antiSMASH. In some cases the gene clusters from ICC1 and ICC4 showed high levels of identity to known BGCs; in others, gene cluster identification was done based on the expected biosynthesis of the compound and the presence of the requisite genes required to install that functionality in the gene cluster. Two examples are described below.

### 2′, 5′ – Dimethoxyflavone and Nordentatin

**FIGURE 5:**
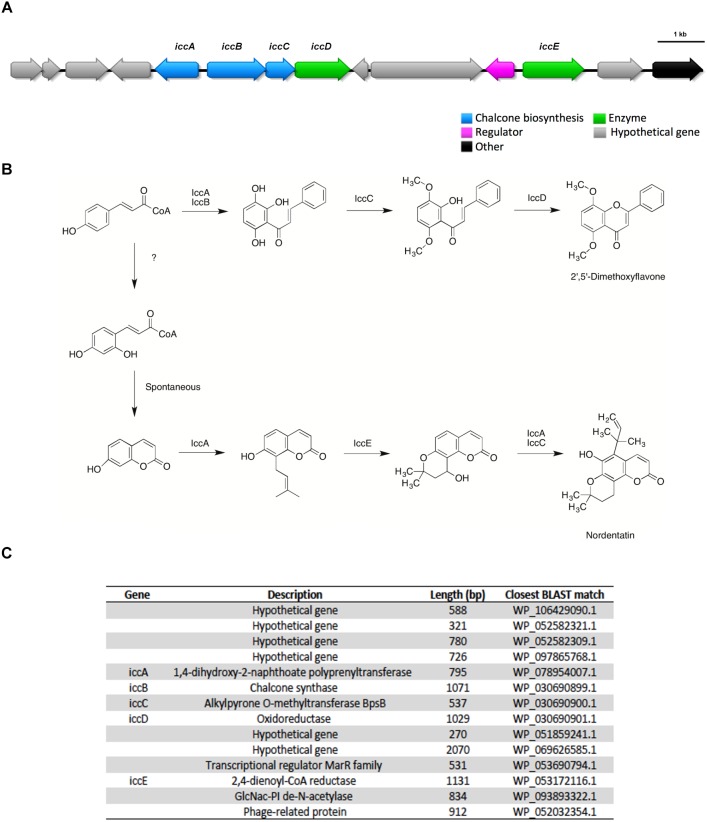
The type III polyketides 2′, 5′ – dimethoxyflavone and nordentatin were identified in metabolomic analysis of culture broth and a potential putative gene cluster encoding these two compounds was identified by antiSMASH analysis of the genomes of both *Streptomyces* sp. strains ICC1 and ICC4. **(A)** Biosynthetic gene cluster for both type III polyketides. **(B)** Proposed mechanism for their biosynthesis. **(C)** List of genes in the cluster, annotation, length and similarity based on BLAST library alignment.

2′, 5′ – dimethoxyflavone and nordentatin, both type III polyketides were identified by GNPS from the UHPLC-HRMS data of the aqueous and *n*-butanolic fractions of *Streptomyces* sp. ICC1, respectively. A BGC likely encoding these type III polyketides was identified from the antiSMASH predictions for both strains. A manual genome annotation analysis identified this as the only type III PKS gene cluster in the genome, consistent with the antiSMASH results. Very little information about both molecules is available in the literature, with nordentatin being described as possessing antibacterial activities against Gram-positive and Gram-negative strains (Wu and Furukawa, 1982). Based on the identified gene cluster and the structure of the metabolites, we provide a putative biosynthesis of both compounds from this single gene cluster (Figure 5).

### Diazepinomicin

One of the terpenes gene clusters predicted by antiSMASH possesses a seven gene pathway that is consistent with diazepinomicin biosynthesis. Diazepinomicin contains a very rare core structure and its BGC, to date, has only been identified from the genome of the sponge-associated actinomycete *Micromonospora* sp. DPJ12 genome (McAlpine et al., 2008). Diazepinomicin possesses modest antibacterial activity against Gram-positive strains (Charan et al., 2004).

**FIGURE 6:**
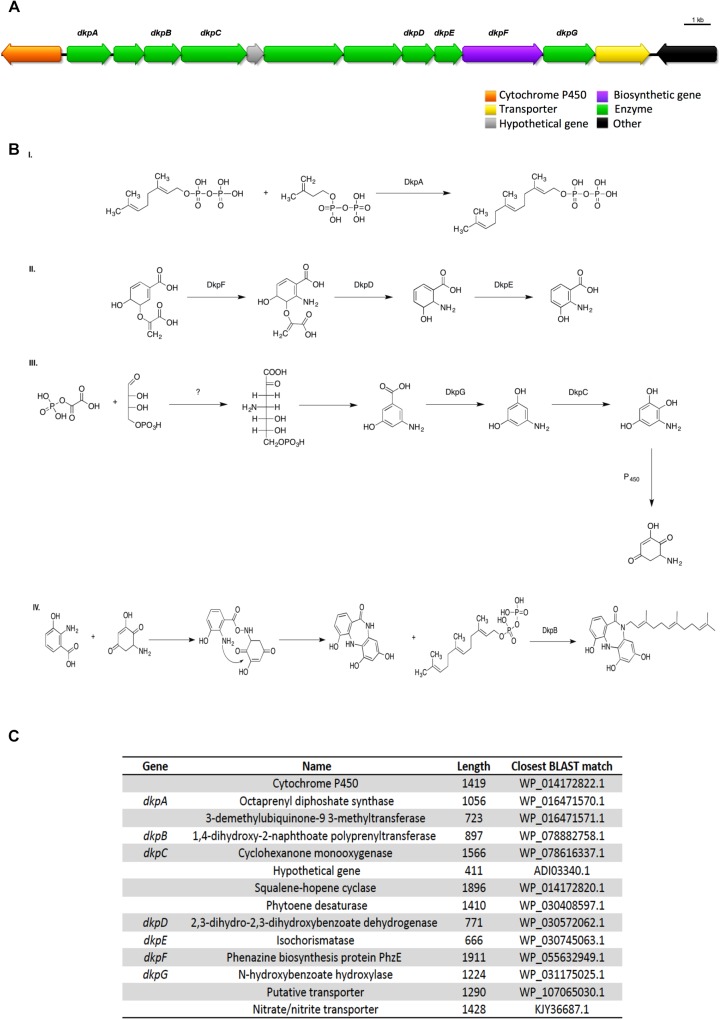
Proposed diazepinomicin biosynthetic pathway from *Streptomyces* sp. strains ICC1 and ICC4. Diazepinomicin was detected in one of the fractions from ICC1. **(A)** biosynthetic gene cluster diazepinomicin. **(B)** Proposed mechanism for diazepinomicin biosynthesis. **(C)** List of genes in the cluster, annotation, length and similarity based on BLAST library alignment.

UHPLC-HRMS analysis of the *n*-butanolic fraction of *Streptomyces* sp. ICC1 identified the presence of the diazepinomicin core structure. A putative biosynthesis mechanism based on the genes identified in the genome and the proposed biosynthesis by McAlpine et al. (2008) is shown on Figure 6. Key aspects of this assignment are DkpF, DkpD, and DkpE, which we propose are responsible for the formation of 3-hydroxyanthranilic acid, one of two key building blocks for diazepinomicin biosynthesis. This is fully consistent with the biosynthetic pathway identified by McAlpine et al. (2008). Similarly DkpG, DkpC and one of the oxidases encoded in the pathway are proposed to be responsible for conversion of 3-amino-5-hydroxybenzoic acid into 2-amino-6-hydroxy [1,4]benzoquinone. This is also fully consistent with the *Micromonospora* biosynthetic mechanism. While the ICC1 and ICC4 gene clusters encode the key functionality for diazepinomicin biosynthesis, they show little homology or synteny with the sequenced *Micromonospora* pathway, suggesting that they may have evolved separately.

## Discussion

The Iron Curtain Cave is a carbonate cave located near Chilliwack, British Columbia, Canada. The cave was discovered by Rob Wall in 1993 and has a unique environment with high iron content sediment and there are cave decorations/speleothems such as soda straws, popcorns, bacon strips (curtain), stalactites, stalagmites, and flowstone structures throughout, hence the name (Ghosh et al., 2017). The unique reddish coloration in cave decorations and sediments originate from the iron-rich environment; it also has a clay consistency indicating higher levels of moisture as evident by a number of water pools in the cave. Access to this cave has been very limited with locked gates. The cave custodian, Rob Wall, must be contacted and consulted prior to any access. Within the cave, a specific dedicated path is used to preserve the features and habitat of the cave. In our study, both the S*treptomyces* sp. ICC1 and ICC4 were isolated from Point 1 (Connection Room, near “Squeeze” dig around and “Looking Pool”), which is 22.47 m from the entrance of the cave (Ghosh et al., 2017).

Our findings were consistent with other studies showing that limestone caves are inhabited by a multitude of Actinomycetes (Groth et al., 1999; Northup et al., 2003, 2011) with antimicrobial properties (Kieser et al., 2000). For instance, a study on the moonmilk sample of Grotte des Collemboles in Belgium retrieved 78 *Streptomyces* isolates that exhibited varied pigmentation patterns and morphological features. All these strains when tested for antimicrobial properties exhibited strong inhibitory effect on Gram-positive, Gram-negative bacteria, and fungi and also the multidrug resistant *Rasamsonia argillacea* (Maciejewska et al., 2016). Extreme habitat microbiota remain as an interesting source of biological and chemical diversity.

Our genome analysis showed significant similarities between the genomes of ICC1, ICC4, and *S. lavendulae*. Blast analysis showed all three genomes to have high level of identity and subsystem analysis showed the cave strains to possess a similar number of subsystems with similar numbers of ORFs in each subsystem. The secondary metabolite analysis using antiSMASH resulted in a similar number and diversity of BGCs identified on the ICC1 and ICC4 genomes. Interestingly, while the overall genomes of ICC1, ICC4 and *S. lavendulae* are highly similar, ICC1 and ICC4 have approximately 40% more potential BGCs than *S. lavendulae* (37 clusters for ICC1, 35 for ICC4, and 26 for *S. lavendulae*). These observations are consistent with little to no adaptation of the hypogean strains versus their terrestrial counterpart and are consistent with a limited restriction on dispersal in structuring microbial communities in the Iron Curtain Cave.

Highly related strains that differ in their secondary metabolite potential can exhibit functional adaptation. For example the marine actinomycetes *Salinispora tropica* and *Salinispora arenicola* share 87.2% identity among their 3606 orthologs. Their genomes differ primarily in 21 genomic islands, many of which are enriched with large clusters of genes devoted to the biosynthesis of secondary metabolites (Penn et al., 2009). In total *S. tropica* was found to have 19 BGCs and *S. arenicola* 30. Biogeographical characterization showed *S. arenicola* to have a cosmopolitan distribution whereas *S. tropica* was shown to be highly restricted in its distribution (Jensen and Mafnas, 2006). The correlation between enhanced biogeographical distribution and enhanced secondary metabolite potential in highly related actinomycetes suggest that this functional trait may be a driver of ecological diversification in closely related species and may account for the fitness of ICC1 and ICC4 versus *S. lavendulae* in the Iron Curtain Cave.

Through a combination of UHPLC-HRMS experiments and bioinformatics analysis of the genome, a number of secondary metabolites were directly associated with ICC1 and ICC4. Surprisingly for the cave *Streptomyces* strains, relatively few polyketide and non-ribosomal peptide BGCs were detected in the genomes. The most commonly associated secondary metabolite pathway were those annotated as terpene gene clusters. In our UHPLC-HRMS datasets analyzed by GNPS, small and low complexity dipeptides and lipids composed the majority of hits.

Among the UHPLC-HRMS identified compounds, three diketopiperazines were observed, cyclo (Leu- Pro), cyclo (Pro-Val) and cyclo (4-hydroxyPro)-Leu]). Cyclic peptide biosynthesis can occur via dedicated cyclodipeptide synthases which link and cyclize the two amino acids from two aminoacyl-tRNAs to generate the diketopiperazine (Belin et al., 2012), or via NRPSs. In our study the genetic origin of the observed diketopiperazines is still unclear, but the lack of a discrete cyclodipeptide synthase and the presence of apparent incomplete or short NRPS encoding gene suggests these may be NRPS-derived metabolites. Diketopiperazines are commonly seen in bacteria, though were often disregarded as by-products (Da Silva et al., 2017) due to their small size and lack of complexity. With an array of activities, ranging from communication and quorum-sensing to selective antifungal and antibacterial molecules (Belin et al., 2012), they may play a role in the biology of ICC1 and ICC4.

Gene clusters encoding siderophore biosynthesis are commonly found in bacterial genomes. Iron is an essential nutrient for all bacteria. However, due to its low abundance in some environments, siderophore secondary metabolites with high affinity for iron, are often produced by the bacteria to scavenge iron from the environment. While the Iron Curtain cave is rich in iron deposits, ferric iron has low solubility (10^-17^ M at pH 7) in aqueous environments (Andrews et al., 2003). Thus coelichelin, the siderophore whose BGC was identified in both ICC1 and ICC4, may play a role in resolublizing iron for uptake. Intriguingly, coelichelin may also play a role in zinc homeostasis as the biosynthesis of the nearly structural identical compound coelibactin in *S. coelicolor* is regulated by Zn(II) levels (Hesketh et al., 2009).

The two coumarins and diazepinomicin were both identified by MS analysis of *Streptomyces* sp. ICC1 *n*-butanolic and aqueous extracts and were predicted through genome mining. The cluster prediction was non-obvious for diazepinomicin as it differed substantially from the characterized *Micromonospora* gene cluster. This highlights the importance of manually checking annotations derived from genome mining as well as the potential for highly divergent gene clusters encoding the identical molecule particularly in the case where one of the organisms is a facultative or obligate symbiont.

## Conclusion

Caves are environments characterized by low levels of nutrients and limited physical conditions. Nevertheless, these unique habitats harbor a diverse microorganism community. Our work examines and correlates the genomes and metabolomes of two cultivatable isolates from the Iron Curtain Cave. We show that the genomes of these isolates are highly homologous to a known terrestrial *Streptomyces* species, *S. lavendulae*. A key area of difference between the ICC isolates and *S. lavendulae* are the secondary metabolites encoded in the genome. Using UHPLC-HRMS, we were able to detect predicted secondary metabolites as well as metabolites whose biosynthetic origin is unknown. We propose that this metabolic potential may improves the fitness ICC1 and ICC4 in the Iron Curtain Cave.

We propose that the expanded metabolic potential of ICC1 and ICC4 improves their fitness in unique microenvironment of the Iron Curtin cave. Thus we suggest that while cave environments may always not possess significant new microbial strain diversity in this study, they remain an exciting new opportunity for bioprospecting due to the role secondary metabolite biosynthesis plays in increasing fitness in these demanding environments.

## Author Contributions

NC and CB obtained funding, conceived the study and designed the experiments. JG, SG, and AS conducted the experiments. DO designed the MS workflow. AS was instrumental in collecting the MS data sets. JG and SG drafted the manuscript. All authors read and edited the manuscript.

## Conflict of Interest Statement

The authors declare that the research was conducted in the absence of any commercial or financial relationships that could be construed as a potential conflict of interest.

## Supplementary Material

TABLE S1 GNPS Molecular Networking compound identification of n-butanolic and aqueous extracts of *Streptomyces* sp. strain ICC1.
